# Gain-of-function mutation in the voltage-gated potassium channel gene *KCNQ1* and glucose-stimulated hypoinsulinemia - case report

**DOI:** 10.1186/s12902-020-0513-x

**Published:** 2020-03-13

**Authors:** Jinyi Zhang, Christian R. Juhl, Louise Hylten-Cavallius, Morten Salling-Olsen, Allan Linneberg, Jens Juul Holst, Torben Hansen, Jørgen K. Kanters, Signe S. Torekov

**Affiliations:** 1 Department of Biomedical Sciences, Faculty of Health and Medical Sciences, University of Copenhagen, Copenhagen, Denmark; 2 Novo Nordisk Foundation Center for Basic Metabolic Research, Faculty of Health and Medical Sciences, University of Copenhagen, Copenhagen, Denmark; 3 Laboratory for Molecular Cardiology, Department of Cardiology, Copenhagen University Hospital, Rigshospitalet, Copenhagen, Denmark; 4 Center for Clinical Research and Prevention, Bispebjerg and Frederiksberg Hospital, The Capital Region of Denmark, Copenhagen, Denmark

**Keywords:** Voltage-gated-potassium channels, Gain-of-function, Glucose metabolism

## Abstract

**Background:**

The voltage-gated potassium channel Kv7.1 encoded by *KCNQ1* is located in both cardiac myocytes and insulin producing beta cells. Loss-of-function mutations in *KCNQ1* causes long QT syndrome along with glucose-stimulated hyperinsulinemia, increased C-peptide and postprandial hypoglycemia. The *KCNE1* protein modulates Kv7.1 in cardiac myocytes, but is not expressed in beta cells. Gain-of-function mutations in *KCNQ1* and *KCNE1* shorten the action potential duration in cardiac myocytes, but their effect on beta cells and insulin secretion is unknown.

**Case presentation:**

Two patients with atrial fibrillation due to gain-of-function mutations in *KCNQ1* (R670K) and *KCNE1* (G60D) were BMI-, age-, and sex-matched to six control participants and underwent a 6-h oral glucose tolerance test (OGTT). During the OGTT, the *KCNQ1* gain-of-function mutation carrier had 86% lower C-peptide response after glucose stimulation compared with matched control participants (iAUC_360min_ = 34 pmol/l*min VS iAUC_360min_ = 246 ± 71 pmol/l*min). The *KCNE1* gain-of-function mutation carrier had normal C-peptide levels.

**Conclusions:**

This case story presents a patient with a gain-of-function mutation *KCNQ1* R670K with low glucose-stimulated C-peptide secretion, additionally suggesting involvement of the voltage-gated potassium channel *KCNQ1* in glucose-stimulated insulin regulation.

## Background

Impaired function of the voltage-gated potassium channels Kv7.1 (encoded by *KCNQ1*) and Kv11.1 (encoded by *KCNH2*), caused by inheritable mutations or drugs leads to long QT syndrome (LQTS) characterised by malignant cardiac arrhythmias [1]. Moreover, inhibition of Kv7.1 and Kv11.1 increases the glucose-stimulated insulin and C-peptide secretion from the pancreatic beta cells and increases glucagon-like peptide (GLP)-1 in mice [2, 3] and we have previously shown that patients with LQT1 (due to loss of function mutations in *KCNQ1*) and LQT2 (due to loss of function mutations in *KCNH2*) have glucose-stimulated hyperinsulinemia and postprandial hypoglycaemia [2, 4].

*KCNQ1* is expressed in human beta cells [5] and blockage of the channel increases glucose- stimulated insulin secretion [3], and its overexpression impairs glucose-stimulated insulin secretion [6]. *KCNE1* encodes a human potassium channel accessory (β) subunit, and modulates Kv7.1 in cardiomyocytes, but does not seem to be expressed in beta cells [7]. Gain-of-function mutations in either *KCNQ1* or *KCNE1* genes shorten the action potential duration and effective refractory period in cardiomyocytes, increasing the risk of atrial fibrillation (AF) [8, 9].

In this case study, we investigated glucose-stimulated hormone secretion in two patients with AF due to confirmed gain-of-function mutations *KCNQ1* R670K and *KCNE1* G60D, respectively*.* Expression in *Xenopus laevis* oocytes of *KCNQ1* R670K or Kv7.1 co-expressed with *KCNE1* G60D resulted in larger current amplitudes compared with wildtype, confirming a gain-of-function phenotype [8, 9] of the mutations.

We hypothesized that patients with a *KCNQ1* gain of function mutation would have decreased glucose-induced insulin and C-peptide secretion, whereas patients with gain of function mutations in *KCNE1* would be expected to have normal insulin and C-peptide secretion upon glucose stimulation.

## Case presentation

We present two patients with AF who are confirmed heterozygous gain-of-function mutations carriers, recruited from the outpatient clinic at Department of Cardiology, Rigshospitalet, Denmark. One patient had persistent AF and carried the KCNQ1 R670K mutation, while the other patient had paroxysmal AF and carried the KCNE1 G60D mutation. Neither patients had echocardiography abnormalities. For comparison with normal glucose metabolism and ECG profiles, six control participants were BMI, age and sex-matched with the AF patients recruited from the Danish populations studies Inter99, Health 2006, Health 2010 and DanFund studies.

The methods used for the investigations and sample analyzing were previously detalied described in [2]. Below follows a condensed version. The patients and control participants each underwent a 6-h oral glucose tolerance test (OGGT) after overnight fasting. The patients did not take medication the morning before the examination. In a resting state, baseline ECG and blood samples were taken 15, 10 and 0 min before ingestion of a standard 75 g glucose solution. During the following 6 h, ECG and blood samples were taken every 15 min for the first hour and then every 30 min for the remaining 5 h.

Height and weight were measured and BMI calculated as height (m) / weight (kg)^2. Fat percentage was measured using bioimpedance (Biodynamics BIA 310e, Biodynamics, Seattle, WA).

Plasma glucose was measured using an automated Vitros 5.1 FS/5600 analyzer (Ortho Clinical Diagnostics, lower quantitation limit: 19.8 mg/Dl, intra- and interassay coefficients of variation: 0.025). Serum C-peptide was measured using an automated Cobas e411 analyzer (Roche) (analytic detection limit: 1.4–3 pmol/L, intra- and interassay coefficients of variation < 0.04 and < 0.025 respectively. Plasma glucagon and GLP-1 were measured using validated radioimmunoassays with a detection limit < 1 pmol/L [10].

12-lead ECGs were recorded in a resting supine position using a MAC1600 ECG machine (GE Healthcare, Milwaukee, WI). Bazett’s formula (QTcB = QT/(RR)1/2) and Fridericia’s formula (QTcF = QT/(RR)1/3) were used to correct the QT interval by heart rate (RR).

For continuous glucose monitoring (CGM), the participants agreed to wear an iPro2 CGM (Medtronic, Watford, U.K.) between 3 and 7 days. During this period each meal was noted with time and meal composition.

HOMA-IR index was calculated as (fasting glucose (mmol/l) x fasting insulin (μIU/ml))/22.5. HOMA-Beta was calculated (20 x fasting insulin (μIU/ml))/(fasting glucose (mmol/ml) - 3.5).

Findings: There were no differences in HbA1c, fasting hemoglobin, fasting total cholesterol or fasting creatinine between the patients and the corresponding control participants. None of them had HbA1c levels ≥48 mmol/mol (Table 1).

**Table 1:** Subject characteristics of the *KCNQ1* R670K carrier (*KCNQ1*) and *KCNE1* G60D carrier (*KCNE1*) and their BMI, sex and age matched control participants. Subject characteristics and fasting baseline of glucose and hormone levels of the mutation carriers and their matched control participants (*n* = 6), and baseline of ECG (*n* = 4), mean ± SD

	*KCNQ1*	*KCNE1*	Control
Sex	Male	Male	Male
Age	48	49	48 ± 1
BMI (kg/m^2^)	28.0	20.7	24.7 ± 4.3
Fat percentage	25.6	14.2	21.8 ± 5.8
HbA1c (mmol/mol)	31.0	34.0	34.0 ± 3.6
Hgb (mmol/l)	9.0	9.1	8.9 ± 0.8
Cholesterol (mmol/l)	4.0	5.9	4.7 ± 0.7
Creatinine (μmol/l)	62	71	77 ± 7
ECG characteristics in fasting state			
QTcB (ms)	434.6	449.5	410.5 ± 26.8
QTcF (ms)	427.8	458.8	417.9 ± 17.2
Heart rate (beats/min.)	66.4	53.5	54.7 ± 10.7
Fasting glucose and hormone levels			
Serum C-peptide (pmol/l)	774	403	576 ± 301
Serum Insulin (pmol/l)	88	21	42 ± 47
Plasma total GIP (pmol/l)	15	7	9 ± 2
Plasma total GLP-1 (pmol/l)	18	14	12 ± 2
Plasma Glucagon (pmol/l)	7	3	7 ± 4
Plasma Glucose (mmol/l)	5.6	5.2	5.1 ± 0.4

**Fig. 1:**
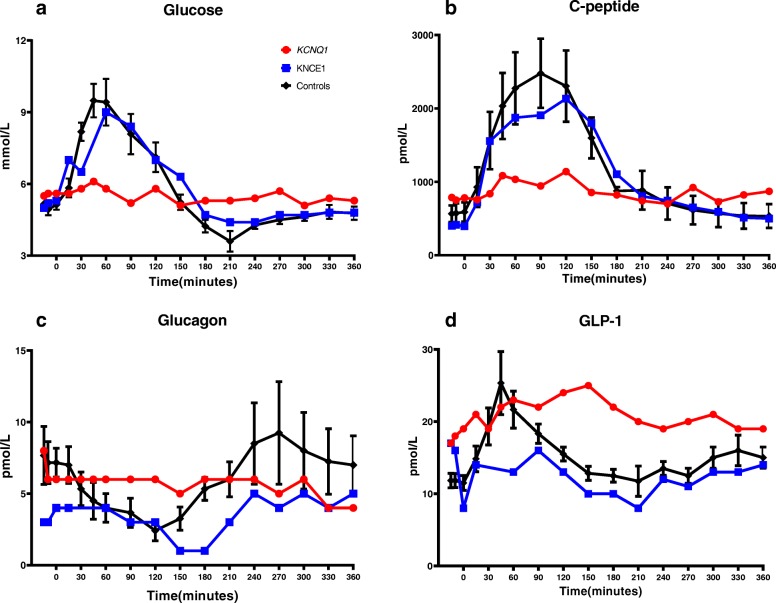
Plasma glucose and hormone responses to oral glucose tolerance test (OGTT). Six hour OGTT and results from Glucose (**a**), C-peptide (**b**), Glucagon (**c**), and GLP-1 (**d**) of the *KCNQ1* R670K carrier (*KCNQ1*) and *KCNE1* G60D carrier (*KCNE1*) and their BMI, sex and age matched control participants (*n* = 6), mean ± SEM

At fasting state, the *KCNQ1* R670K carrier presents with slightly higher fasting insulin levels (but still within the levels observed in the control participants (insulin 88 vs range 14–137 pmol/L and C-peptide 774 vs 338–1226 pmol/L) and therefore increased HOMA-IR (3.1 vs 1.5 ± 1.2) and HOMA-Beta (123% vs 70 ± 55% compared to control participants). In contrast, during glucose stimulation the *KCNQ1* R670K carrier had a markedly blunted C-peptide response and lower glucose levels compared to control participants and the *KCNE1* G60D carrier (Fig. 1 and S1). The glucose-stimulated GLP-1 response was also blunted in the KCNQ1 GOF patient compared to control participants, whereas glucagon response did not differ among the examined participants (Fig. 1). During CGM for 3–7 days, the *KCNQ1* mutation carrier had lower increase in blood glucose levels within 1 h after carbohydrate rich meals (mean increase of 0.8 ± 0.6 mmol/l), compared to both matched controls and the *KCNE1* mutation carrier (mean increase of 1.5 ± 0.4 mmol/L and 1.7 ± 0.5 mmol/L, respectively) (Fig. S2).

**Fig. 2:**
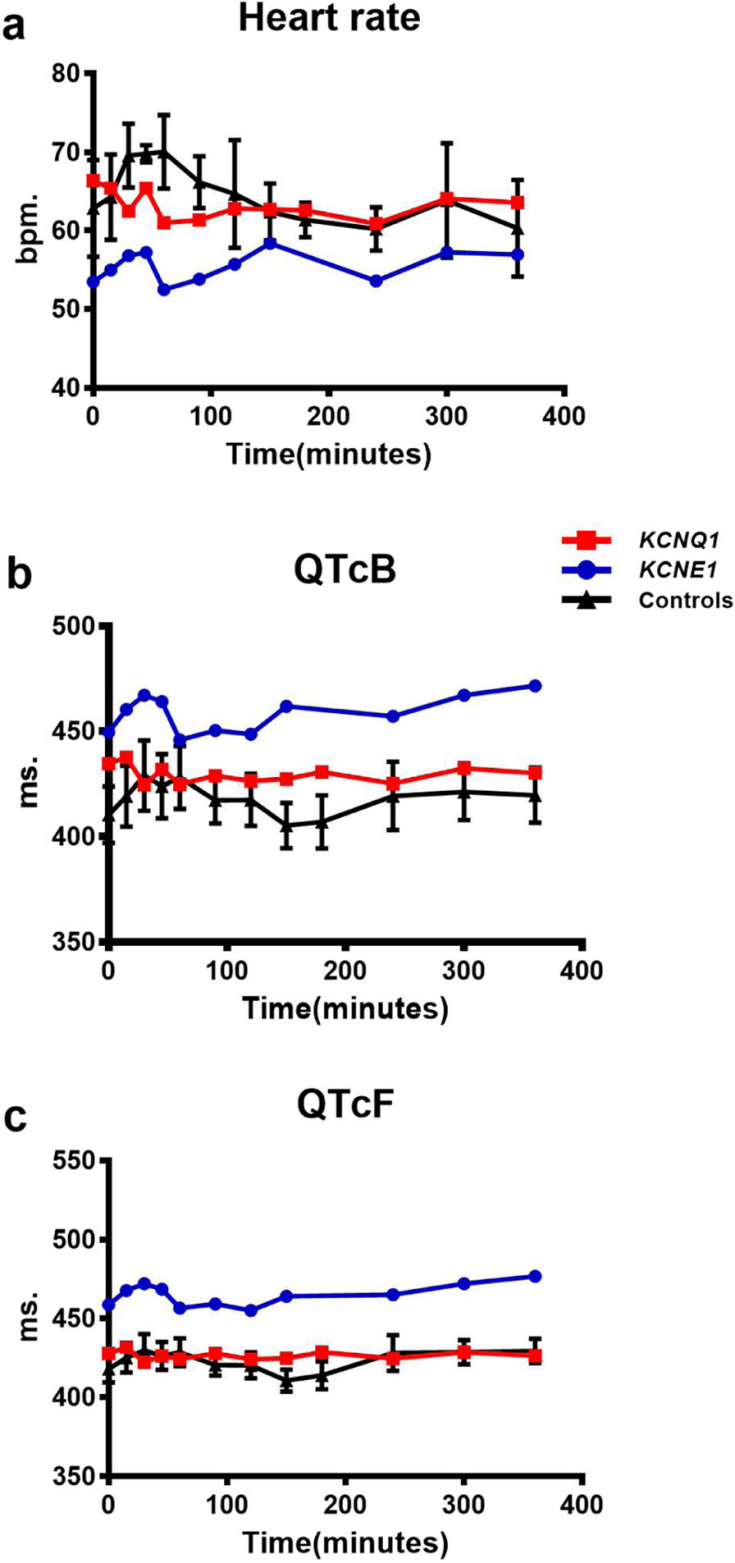
Results from the ECG measurement during the oral glucose tolerance test (OGTT). Six hour OGTT and results from Heart rate (**a**), QTcB (**b**) and QTcF (**c**) of the *KCNQ1* R670K carrier (*KCNQ1*) and *KCNE1* G60D carrier (*KCNE1*) and their BMI, sex and age matched control participants (*n* = 4), means ± SEM

The two patients had similar cardiac profiles as previously reported [8, 9] (Fig. 2).

## Discussion and conclusions

We previously identified that patients with loss-of-function mutations in *KCNQ1* have increased glucose-stimulated C-peptide and insulin secretion, but normal fasting levels [4]. Kv7.1 is expressed in human beta cells and participates in depolarization-evoked insulin exocytosis [5]. In this case study of a patient with gain of function mutation in *KCNQ1*, we observed a lower glucose-stimulated C-peptide and GLP-1 response compared to matched control participants. This may be due to shorter repolarization duration in beta and L-cells cells, similar to what has been observed for this variant with shortened action potential duration in *Xenopus laevis* oocytes [8, 9]. Furthermore, this observation is in agreement with studies of overexpression of *KCNQ1* showing an increased glucose stimulated K^+^ current and impaired and limited glucose stimulated insulin secretion from beta-cells [6]. The *KCNQ1* gain of function patient had a low increase in glucose level after glucose ingestion both during OGTT and during 7 days continuous glucose monitoring, even though the C-peptide in response to glucose stimulation were low. The patient reported that he bicycled more than 20 km every weekday, which may explain the high insulin sensitivity in the glucose-stimulated state of the patient making him able to compensate for the low C-peptide levels by increased glucose uptake in the muscles. Intron variants in *KCNQ1* associated with increased risk of type 2 diabetes in genome wide association studies seem to increase the function of *KCNQ1*, whereas siRNA silencing decrease *KCNQ1* function and increase exocytosis of insulin [5].

Thus, with time and without exercise the *KCNQ1* gain of function patient may be in risk of type 2 diabetes.

We examined another AF patient with gain of function mutation in *KCNE1* that is not expressed in pancreas [7], this patient had a similar C-peptide response compared to the matched control participants. Hence *KCNE1* does not seem to function as a beta subunit of *KCNQ1* in human beta-cells.

Although limited by the very modest sample size, this study provides additional suggestions of the involvement of the voltage-gated potassium channel *KCNQ1* in insulin regulation.

## Supplementary information

**Additional file 1: Figure S1.** Plasma glucose and C-peptide responses to oral glucose ingestion in the *KCNQ1* R670K carrier (*KCNQ1*) and *KCNE1* G60D carrier (*KCNE1*) and their BMI, sex and age matched control participants. Control to KCNQ1 (R670K) (*n* = 2, men, BMI = 26.8 ± 0.7, age = 49.4 ± 2.3, fat% = 23.9 ± 3.5). Control to KCNE1 (G60D) (n = 2, men, BMI = 19.6 ± 1.3, age = 49.4 ± 0.6, fat% = 16.8 ± 6.6). **Figure S2.** Results from 3 to 7 day continuous glucose monitors (CGM). Increase of blood glucose levels within 1 h after carbohydrate rich meals(a) and the mean glucose levels for during the whole period(b) from the *KCNQ1* (red) R670K carrier (*KCNQ1*) and *KCNE1* G60D carrier (*KCNE1*) (blue) and their matched control participants, means ± SEM.

## Data Availability

The datasets generated during and/or analyzed during the current study are not publicly available but are available from the corresponding author on reasonable.

## Abbreviations

AF: Atrial fibrillation
LQTS: Long QT syndrome
OGTT: Oral glucose tolerance test
