# Supplementary material for: Gain-of-function mutation in the voltage-gated potassium channel gene KCNQ1 and glucose-stimulated hypoinsulinemia - case report
Source: BMC Endocr Disord. 2020 Mar 13;20:38. doi: 10.1186/s12902-020-0513-x (PMC7069191; doi:10.1186/s12902-020-0513-x)
Supplement: Supplementary file 1 — Additional file 1: Figure S1. Plasma glucose and C-peptide responses to oral glucose ingestion in the KCNQ1 R670K carrier (KCNQ1) and KCNE1 G60D carrier (KCNE1) and their BMI, sex and age matched control participants. Control to KCNQ1 (R670K) (n = 2, men, BMI = 26.8 ± 0.7, age = 49.4 ± 2.3, fat% = 23.9 ± 3.5). Control to KCNE1 (G60D) (n = 2, men, BMI = 19.6 ± 1.3, age = 49.4 ± 0.6, fat% = 16.8 ± 6.6). Figure S2. Results from 3 to 7 day continuous glucose monitors (CGM). Increase of blood glucose levels within 1 h after carbohydrate rich meals(a) and the mean glucose levels for during the whole period(b) from the KCNQ1 (red) R670K carrier (KCNQ1) and KCNE1 G60D carrier (KCNE1) (blue) and their matched control participants, means ± SEM. [file 12902_2020_513_MOESM1_ESM.docx]

**Supplements**

**Figure S1.** **Plasma glucose and C-peptide responses to oral glucose ingestion in the *KCNQ1* R670K carrier (*KCNQ1*) and *KCNE1* G60D carrier (*KCNE1*) and their BMI, sex and age matched control participants.** Control to KCNQ1 (R670K) (n=2, men, BMI=26.8±0.7, age= 49.4±2.3, fat%=23.9±3.5). Control to KCNE1 (G60D) (n=2, men, BMI=19.6±1.3, age= 49.4±0.6, fat%=16.8±6.6)

**Figure S2 Results from 3-7 day continuous glucose monitors (CGM).** Increase of blood glucose levels within 1hr after carbohydrate rich meals(a) and the mean glucose levels for during the whole period(b) from the *KCNQ1* (red) R670K carrier (*KCNQ1*) and *KCNE1* G60D carrier (*KCNE1*) (blue) and their matched control participants, means ± SEM.

**
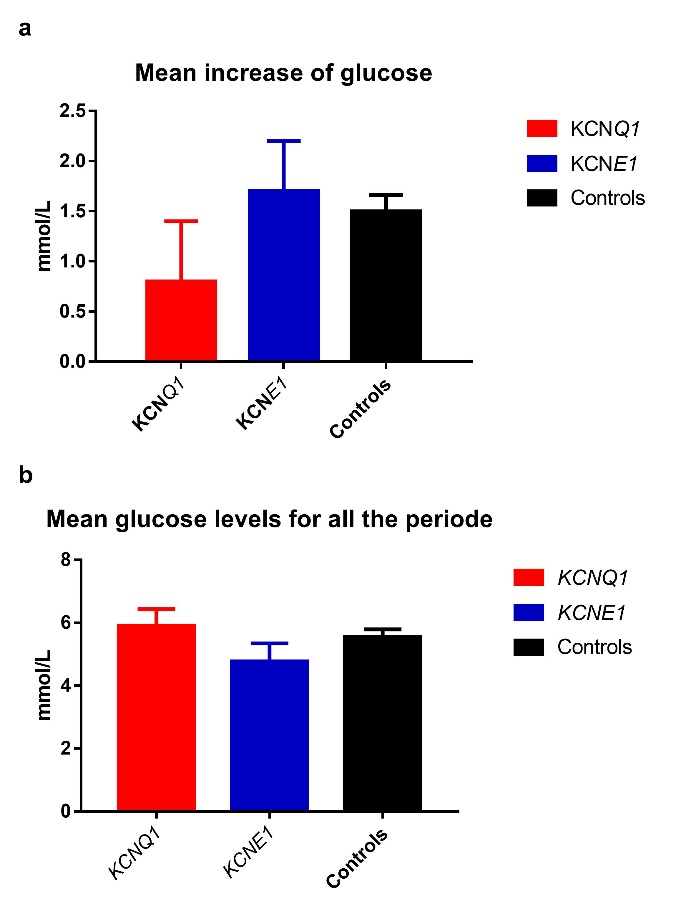
**
